# Quality of Life Among Breast Cancer Survivors Treated With Modified Radical Mastectomy: A Cross-Sectional Study Using the European Organisation for Research and Treatment of Cancer (EORTC) QLQ-BR42 Questionnaire

**DOI:** 10.7759/cureus.89999

**Published:** 2025-08-13

**Authors:** Revathy Nair ES, Hemanth S Ghalige, Vinaya Gopalaswamy, Arunkumar Tukaram, Shwetha Subbaramaiah

**Affiliations:** 1 Surgery, Employees' State Insurance Corporation Medical College (ESIC MC) and Post Graduate Institute of Medical Sciences and Research (PGIMSR), Bengaluru, IND; 2 Internal Medicine, Employees' State Insurance Corporation Medical College (ESIC MC) and Post Graduate Institute of Medical Sciences and Research (PGIMSR), Bengaluru, IND; 3 Medical Oncology, Employees' State Insurance Corporation Medical College (ESIC MC) and Post Graduate Institute of Medical Sciences and Research (PGIMSR), Bengaluru, IND

**Keywords:** carcinoma of breast, eortc qlq-br42, modified radical mastectomy (mrm), quality of life (qol), survivorship needs

## Abstract

Introduction

In India, breast cancer is often diagnosed at advanced stages, leading to a high proportion of patients undergoing modified radical mastectomy (MRM) as the primary surgical treatment. While effective, MRM is associated with significant long-term physical and psychosocial morbidity. This study aimed to assess the quality of life (QoL) of breast cancer survivors treated with MRM in a tertiary care center, utilizing the updated European Organisation for Research and Treatment of Cancer (EORTC) QLQ-BR42 questionnaire.

Methods

A cross-sectional observational study was conducted at the Employees’ State Insurance Corporation Medical College (ESIC MC) and Post Graduate Institute of Medical Sciences and Research (PGIMSR), Bengaluru, from September 2024 to June 2025. A consecutive sample of 300 female breast cancer survivors who had undergone MRM and completed adjuvant therapy was recruited. Data were collected via face-to-face interviews using EORTC QLQ-BR42 modules. QoL scores were calculated and transformed to a 0-100 scale. Group comparisons were performed using independent t-tests and ANOVA to assess differences in QoL between patients who received single modality (adjuvant chemotherapy alone) and those who received dual modality (adjuvant chemotherapy and radiotherapy).

Results

A total of 300 participants were included, with a mean age of 52.4 ± 10.6 years. All had undergone MRM, with 66% (n = 198) receiving dual modality and 34% (n = 102) receiving single modality treatment. The study found high mean linearized scores for body image satisfaction (79.77 ± 8.99) and breast satisfaction (70.00 ± 8.50). However, the future perspective domain was moderately affected (62.00 ± 15.00). Symptom domains showed that breast symptoms (55.68 ± 24.90) and arm symptoms (24.77 ± 10.20) had the highest symptom burden. Sexual health was the most severely impaired aspect, with a mean score of 1.67 for sexual functioning and 0.00 for sexual enjoyment. No statistically significant differences in any QoL domain were observed between the single modality and dual modality treatment groups.

Conclusions

Our study suggests that breast cancer survivors treated with MRM in this cohort report a high degree of satisfaction with their body image but suffer from significant physical symptoms and severely impaired sexual health. The addition of radiotherapy does not appear to significantly impact long-term QoL. The study underscores the need for comprehensive, culturally sensitive, and resource-appropriate supportive care, particularly focusing on sexual health and symptom management, to improve the holistic well-being of these survivors.

## Introduction

Breast cancer is the most frequently diagnosed malignancy among women globally, with over 2.3 million new cases and approximately 685,000 deaths reported in 2020, according to the GLOBOCAN estimates [1]. The global burden of breast cancer continues to rise, with significant improvements in survival outcomes owing to early detection, enhanced treatment modalities, and multidisciplinary care, especially in high-income countries. In these settings, five-year relative survival rates for localized breast cancer often exceed 90% [2], shifting the focus from survival alone to long-term survivorship and quality of life (QoL) [3].

In contrast, breast cancer in low- and middle-income countries (LMICs), including India, is often diagnosed at more advanced stages. Factors contributing to delayed diagnosis include lack of awareness, absence of national screening programs, stigma, and sociocultural barriers [4,5]. Furthermore, limited access to radiotherapy, reconstructive services, and a shortage of trained oncology professionals restrict treatment options, leading to the continued predominance of modified radical mastectomy (MRM) as the surgical treatment of choice [6]. As a result, MRM remains the predominant surgical approach, with breast-conserving surgery (BCS) performed in only 21.3% of patients, compared to the much higher rates seen in Western countries [7].

MRM, while effective, is associated with significant psychosocial and physical morbidity. In high-resource settings, BCS has become the standard of care for early-stage disease, supported by adjuvant radiotherapy and reconstructive techniques that improve cosmetic outcomes and psychological well-being [8]. However, in India, limitations in healthcare infrastructure, availability of radiotherapy, and a lack of psychosocial counselling mean that most patients undergoing MRM do not receive reconstruction or structured postoperative support [9,10].

Results from a recent prospective study of 500 Indian breast cancer patients assessed with the European Organisation for Research and Treatment of Cancer (EORTC) QLQ-BR30 show that, although symptom-related scores tended to improve over time, psychosocial aspects, especially body image and sexual enjoyment, worsened notably by the end of treatment (mean changes of -7.0 and -7.1, respectively) and recovered only partially after six months, remaining below their pretreatment values [11].

These findings underscore the need for a more comprehensive evaluation of survivorship outcomes in the Indian context. While prior Indian studies have explored QoL using older tools such as the EORTC QLQ-BR23, they often lack comprehensive domain coverage and do not reflect current survivorship priorities [12]. The updated EORTC QLQ-BR42 tool, which expands on musculoskeletal, endocrine, and sexual health domains, provides a more robust framework for evaluating QoL among survivors [13].

This study was conducted to assess the QoL among Indian breast cancer survivors treated with MRM using the EORTC QLQ-BR42 instrument. By capturing functional, emotional, symptomatic, and sexual health dimensions and comparing outcomes based on treatment modalities, the study aims to provide insights that may guide culturally sensitive, resource-appropriate survivorship care strategies.

## Materials and methods

Study design and setting

A cross-sectional observational study was carried out at the Department of Medical Oncology, Employees’ State Insurance Corporation Medical College (ESIC MC) and Post Graduate Institute of Medical Sciences and Research (PGIMSR), Bengaluru, India, a tertiary care public sector teaching hospital. The study duration was from September 2024 to June 2025.

Participants

Eligible participants were female breast cancer survivors aged 18 years or older who had undergone MRM as part of their primary treatment, were disease-free and attending regular outpatient follow-up visits at the time of recruitment, and had completed systemic therapy (chemotherapy with or without radiotherapy) at least six months prior to recruitment. Exclusion criteria included recurrent or metastatic disease, concurrent malignancy, significant cognitive impairment, or incomplete primary treatment.

Outcomes

This study primarily aimed to assess the overall QoL and evaluate domain-specific QoL outcomes (including physical, emotional, sexual, and symptomatic dimensions) among breast cancer survivors who have undergone MRM, utilizing the EORTC QLQ-BR42 questionnaire. A secondary objective was to compare QoL scores between patients treated with single modality versus dual modality treatment groups.

Sample size

Based on previous studies [1], a sample size of approximately 296 was required, using a breast cancer prevalence among females of 26% from GLOBOCAN 2020, with a 95% CI and 5% precision. The chosen sample size of 300 participants comfortably exceeds this requirement, providing a robust cohort for comprehensive QoL assessment. This sample size also allows for the exploration and comparison of QoL scores between different treatment modalities as a secondary objective.

Data collection

Participants were recruited consecutively from the oncology clinic. After obtaining informed written consent, data were collected using face-to-face interviews conducted in the participants’ preferred language (Kannada, Hindi, or English). Trained research staff administered the questionnaire and recorded demographic, clinical, and treatment-related details.

QoL instrument

The EORTC QLQ-BR42 is a validated, breast cancer-specific QoL instrument that builds upon the earlier BR23 module [12]. It comprises multiple scales measuring functional outcomes (physical functioning, body image, sexual functioning, and satisfaction), symptoms (systemic therapy side effects, arm symptoms, breast symptoms, musculoskeletal pain, and skin toxicity), and endocrine-related issues. Responses were recorded on 4-point Likert scales and transformed to linear scores ranging from 0 to 100 according to the EORTC Scoring Manual.

Statistical analysis

Descriptive statistics, including means, SDs, and proportions, were calculated for demographic and clinical variables. Functional and symptom domain scores were analyzed separately. Data was checked for normality, and independent t-tests and one-way ANOVA were used for group comparisons, particularly between patients treated with single modality (chemotherapy alone) and those who received dual modality (adjuvant chemotherapy and adjuvant radiotherapy). Statistical significance was set at p < 0.05. All data analyses were conducted using IBM SPSS Statistics for Windows, Version 28.0 (Released 2021; IBM Corp., Armonk, NY, USA).

Ethical considerations

Ethical clearance for the study was obtained from the Institutional Ethics Committee of ESIC Medical College and PGIMSR, Bengaluru. Informed consent was obtained from all participants. Confidentiality and anonymity were strictly maintained throughout the data collection and analysis process.

## Results

A total of 300 female breast cancer survivors were included in the study. The mean age of the cohort was 52.4 ± 10.6 years. Most participants (84%, n = 252) were married, and 60% (n = 180) resided in urban areas. Educational levels were low; 20% (n = 60) had no formal education, and only 20% (n = 60) were graduates. A majority (76%, n = 228) were postmenopausal. None of the participants reported alcohol use.

All participants underwent MRM. Of these, 66% (n = 198) received dual modality treatment, and 34% (n = 102) received single modality treatment. At the time of the survey, all participants were disease-free and had completed their primary treatment at least six months prior.

QoL scores

**Figure 1:**
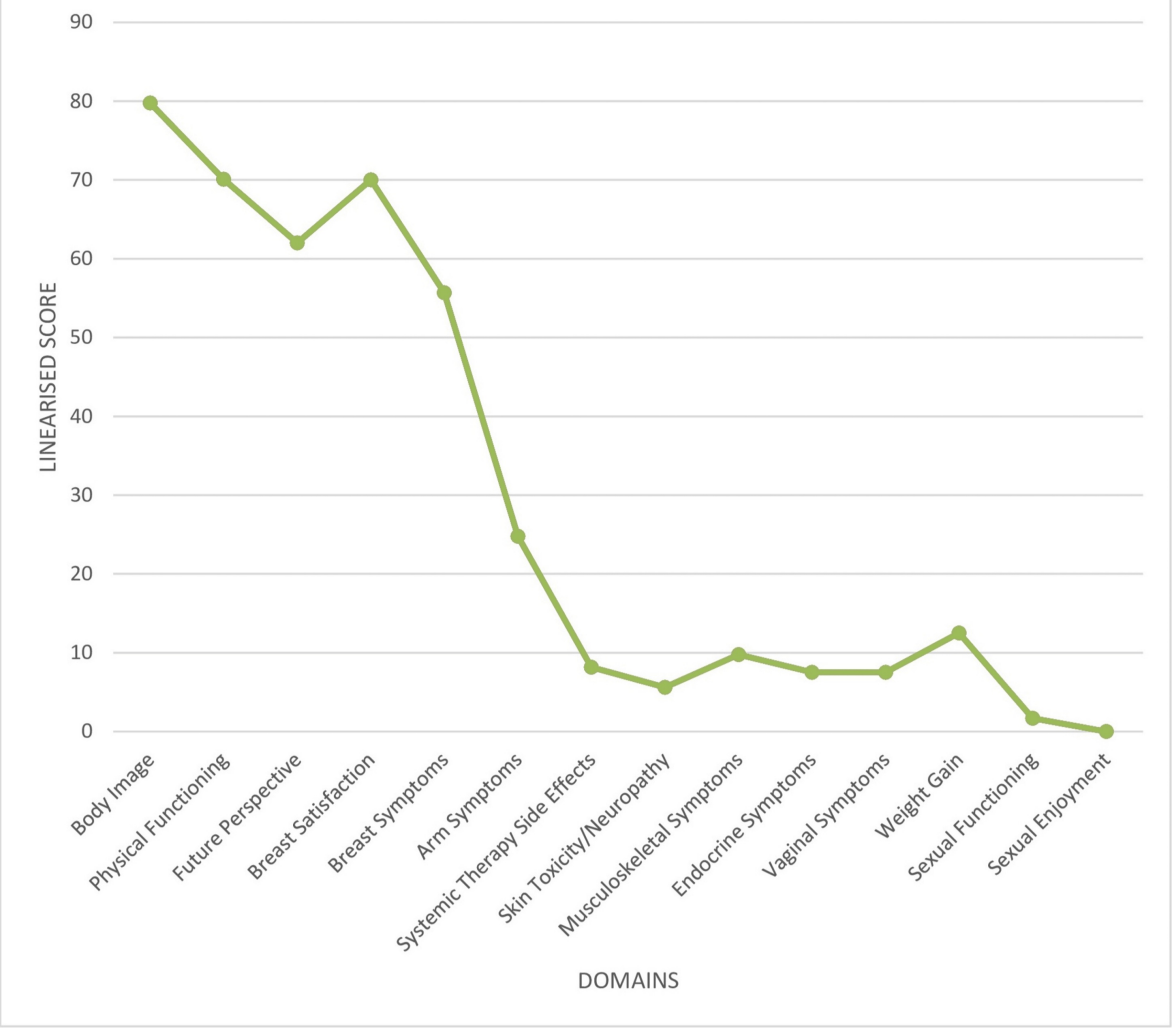
Graphical representation of linear scores of EORTC QLQ-BR42 domain EORTC, European Organisation for Research and Treatment of Cancer

QoL scores indicated high body image satisfaction (mean linearized score: 79.77 ± 8.99) and moderate physical functioning (70.08 ± 20.68). Breast satisfaction was similarly high (70.00 ± 8.50), while the future perspective domain, which evaluates optimism and fears of recurrence, was moderately affected (62.00 ± 15.00) (Figure 1, Table 1).

**Table 1:** EORTC QLQ-BR42 domain scores EORTC, European Organisation for Research and Treatment of Cancer

Domain	Raw score (mean ± SD)	Linearized score (mean ± SD)
Body image	3.19 ± 0.36	79.77 ± 8.99
Physical functioning	3.05 ± 0.62	70.08 ± 20.68
Future perspective	2.50 ± 0.58	62.00 ± 15.00
Breast satisfaction	2.80 ± 0.30	70.00 ± 8.50
Breast symptoms	2.23 ± 0.89	55.68 ± 24.90
Arm symptoms	1.99 ± 0.41	24.77 ± 10.20
Systemic therapy side effects	1.15 ± 0.15	8.15 ± 10.40
Skin toxicity/neuropathy	1.05 ± 0.12	5.60 ± 6.20
Musculoskeletal symptoms	1.12 ± 0.18	9.76 ± 9.39
Endocrine symptoms	1.10 ± 0.22	7.50 ± 8.90
Vaginal symptoms	1.10 ± 0.25	7.50 ± 6.50
Weight gain	1.30 ± 0.28	12.50 ± 7.20
Sexual functioning	1.03	1.67
Sexual enjoyment	1	0

Symptom domains

Symptom domains showed that breast symptoms had the highest burden (55.68 ± 24.90), followed by arm symptoms (24.77 ± 10.20). Other domains, such as systemic therapy side effects (8.15 ± 10.40), musculoskeletal symptoms (9.76 ± 9.39), and endocrine symptoms (7.50 ± 8.90), were relatively mild. Skin toxicity, neuropathy, and weight gain were also infrequent (Table 1, Figure 1).

Sexual health

Sexual health was the most impaired aspect of QoL. Only 10 participants reported sexual activity. When adjusted across the full cohort, the mean linearized score for sexual functioning was 1.67, and sexual enjoyment was 0.00 (Table 1, Figure 1).

Comparison between single modality and dual modality treatment groups

Comparison between single modality and dual modality groups revealed no statistically significant differences across all 13 QoL domains. For example, as depicted in Table 2, body image scores were nearly identical (single modality: 79.93 ± 9.09 vs. dual modality: 79.62 ± 8.92; p = 0.769), and physical functioning scores were also similar (single modality: 69.81 ± 20.54 vs. dual modality: 70.22 ± 20.79; p = 0.821). Symptom scores such as breast symptoms (p = 0.623), arm symptoms (p = 0.249), and endocrine symptoms (p = 0.538) did not differ significantly between groups (Table 2). These results suggest that the addition of radiotherapy to chemotherapy does not significantly alter long-term QoL outcomes in MRM-treated breast cancer survivors.

**Table 2:** Comparison of QoL between single versus dual modality treatment groups QoL, quality of life

Domain	Single modality (mean ± SD)	Dual modality (mean ± SD)	p-value
Functional scales			
Body image	79.93 ± 9.09	79.62 ± 8.92	0.769
Physical functioning	69.81 ± 20.54	70.22 ± 20.79	0.821
Breast satisfaction	54.83 ± 24.91	56.49 ± 24.94	0.565
Symptom scales			
Breast symptoms	54.42 ± 25.10	56.18 ± 24.87	0.623
Arm symptoms	23.70 ± 10.44	25.40 ± 9.97	0.249
Systemic therapy side effects	8.98 ± 8.73	10.50 ± 9.96	0.164
Musculoskeletal pain	9.32 ± 8.97	9.99 ± 9.71	0.684
Endocrine symptoms	7.00 ± 7.98	7.78 ± 9.21	0.538
Skin toxicity/neuropathy	5.49 ± 5.94	5.66 ± 6.30	0.814
Weight gain	11.90 ± 6.92	13.01 ± 7.43	0.254
Vaginal symptoms	7.40 ± 6.22	7.59 ± 6.67	0.818
Emotional and sexual scales			
Future perspective	69.24 ± 22.41	70.88 ± 18.91	0.494
Sexual functioning	50.00 (n = 5)	50.00 (n = 5)	0.242
Sexual enjoyment	0.00 (n = 5)	0.00 (n = 5)	0.242

## Discussion

This study provides a comprehensive evaluation of QoL among 300 Indian breast cancer survivors who underwent MRM, utilizing the recently updated EORTC QLQ-BR42 instrument. Our findings offer crucial insights into survivorship patterns within a resource-constrained setting where MRM remains the predominant surgical approach. While MRM is an effective treatment, our data highlight significant postoperative morbidity, particularly concerning body image, sexual health, and physical functioning. These findings also illuminate the broader psychosocial landscape experienced by survivors in LMICs.

To our knowledge, this is the first study from India to employ the EORTC QLQ-BR42 [13], an instrument recently updated and validated for breast cancer treatments, including items relevant to systemic therapy side effects, neuropathy, and endocrine symptoms. This updated tool offers a more precise and relevant assessment of QoL in this extensively treated population, contributing novel data to the existing literature. Although this was a cross-sectional study, its design is comparable to previous cross-sectional studies that successfully captured similar data, aligning with established methodologies in QoL research [14,15].

Our results indicate high body image satisfaction (mean linearized score: 79.77 ± 8.99) and breast satisfaction (70.00 ± 8.50) among survivors. This finding contrasts with some literature suggesting generally lower body image after MRM compared to BCS [16-19]. For instance, Cherian et al. [17] reported a body image score of 53.5 for MRM patients versus 80.7 for BCS patients, and Curran et al. [18] also noted better body image with BCS. However, our high body image scores might be due to low educational status, adaptive coping mechanisms, or cultural contexts specific to our Indian cohort, where MRM is widely accepted due to limited resources [15,18]. Another longitudinal study from India reported a body image score of 82, which included both BCS and MRM cases [11], aligning more closely with our findings.

Physical functioning in our cohort was moderate (70.08 ± 20.68). This is somewhat lower than the physical functioning score of 76.5 reported for BCS patients by Cherian et al. [17] and 81.5 in a mixed cohort of Indian breast cancer survivors [11]. This suggests that while survivors in our study demonstrated moderate physical capabilities, there might still be room for improvement or targeted interventions to enhance this domain.

The future perspective domain, which assesses optimism and fears of recurrence, was moderately affected (62.00 ± 15.00). Interestingly, Cherian et al. [17] found that emotional functioning and future perspective might be better in MRM patients at one year (future perspective score 62 for MRM vs. 40 for BCS), which aligns with our moderate score, suggesting that despite the physical challenges, a sense of optimism or resilience may prevail in MRM patients.

Regarding symptom burden, breast symptoms (55.68 ± 24.90) and arm symptoms (24.77 ± 10.20) were the most prevalent. This is consistent with common sequelae of MRM, such as lymphedema, pain, and discomfort in the surgical area [11,17]. For comparison, arm symptoms were reported as 65 for BCS and 62 for MRM, and breast symptoms as 41 for BCS and 39 for MRM in one study [17], while another Indian longitudinal study reported lower scores for arm (14.2) and breast (11.7) symptoms [11]. The higher burden in our study may reflect the long-term impact of MRM and the need for ongoing symptom management. Other symptoms like systemic therapy side effects (8.15 ± 10.40), musculoskeletal symptoms (9.76 ± 9.39), and endocrine symptoms (7.50 ± 8.90) were relatively mild, possibly indicating successful management of these treatment-related toxicities in our cohort.

A critical finding of our study is the profound impairment in sexual health, with mean linearized scores of 1.67 for sexual functioning and 0.00 for sexual enjoyment. Only 10 participants reported any sexual activity. This severe impact aligns with research indicating that more extensive surgeries, like mastectomy, often lead to worse sexual health and higher anxiety, which can persist for years [19,20]. Cherian et al. [17] reported that, at one year, sexual enjoyment scores were 7.1 for BCS versus 69.63 for MRM, and sexual function scores were 97.5 for BCS versus 98.4 for MRM. The discrepancy between our findings and Cherian et al. [17] on sexual enjoyment and functioning may be due to cultural differences in discussing and reporting sexual health, age, and postmenopausal status, or variations in the specific items of the QoL instrument used. Psychosexual adjustment is significantly influenced by partner support [21,22], suggesting that lack of perceived support could be a contributing factor to the observed low scores in our cohort.

Importantly, our study found no statistically significant differences in any of the 13 QoL domains when comparing single modality treatment (MRM + chemotherapy) with dual modality treatment (MRM + chemotherapy + radiotherapy). For instance, body image scores were nearly identical (single modality: 79.93 ± 9.09 vs. dual modality: 79.62 ± 8.92; p = 0.769), and physical functioning scores were similar (p = 0.821). This suggests that in our context, the addition of radiotherapy to chemotherapy does not significantly alter long-term QoL outcomes in MRM-treated breast cancer survivors. This finding adds to the limited direct evidence comparing QoL outcomes between these specific treatment approaches. While some studies suggest chemotherapy can improve QoL [23] and radiotherapy can affect physical and functional well-being [16], our results indicate that in settings where MRM with chemotherapy is standard due to resource limitations, adding radiotherapy may not significantly impact the perceived QoL in the long term. This aligns with long-term studies from LMICs suggesting overall QoL can recover over time with no significant differences between surgical groups at five years [16].

Limitations

This study has several limitations. Firstly, its cross-sectional design prevents the establishment of cause-and-effect relationships or the assessment of QoL changes over time. Longitudinal studies would provide a more nuanced understanding of QoL trajectories in this population. Secondly, the study was conducted in a single institution in India, which may limit the generalizability of the findings to other regions or diverse populations with different cultural contexts and healthcare infrastructures. Thirdly, while the EORTC QLQ-BR42 is a comprehensive instrument, QoL is a multifaceted construct, and other unmeasured factors not captured by this tool could influence the results. Finally, the extremely low rates of reported sexual activity and enjoyment might be influenced by cultural sensitivities around discussing sexual health in a survey format, potentially leading to underreporting.

## Conclusions

Our study, leveraging the updated EORTC QLQ-BR42, provides valuable insights into the QoL of Indian breast cancer survivors post-MRM. While body image and breast satisfaction were notably high, sexual health was severely impacted, and breast and arm symptoms presented significant burdens. The lack of significant difference in QoL between single and dual modality treatments suggests that the long-term impact of radiotherapy on QoL, beyond chemotherapy, might be minimal. These findings underscore the need for targeted interventions to address specific domains of QoL, particularly sexual health and symptom management, to enhance the overall well-being of breast cancer survivors in similar resource-constrained settings.
